# *Cucumis metuliferus* Fruit Extract Loaded Acetate Cellulose Coatings for Antioxidant Active Packaging

**DOI:** 10.3390/polym12061248

**Published:** 2020-05-29

**Authors:** Marina Patricia Arrieta, Luan Garrido, Simón Faba, Abel Guarda, María José Galotto, Carol López de Dicastillo

**Affiliations:** 1 Departamento de Química Orgánica, Facultad de Óptica y Optometría, Universidad Complutense de Madrid (UCM), Arcos de Jalón 118, 28037 Madrid, Spain; 2 Center of Innovation in Packaging (Laben), Department of Science and Food Technology, Faculty of Technology, Center for the Development of Nanoscience and Nanotechnology (CEDENNA), Universidad de Santiago de Chile (USACH), 9170201 Santiago, Chile; luan.garrido@usach.cl (L.G.); simon.riverosf@usach.cl (S.F.); abel.guarda@usach.cl (A.G.); maria.galotto@usach.cl (M.J.G.)

**Keywords:** *Cucumis metuliferus*, extraction, antioxidant activity, coating, cellulose acetate, LDPE, bilayer packaging, active packaging

## Abstract

A new active coating was developed by using *Cucumis metuliferus* fruit extract as antioxidant additive with the aim of obtaining an easy way to functionalize low-density polyethylene (LDPE) films for food packaging applications. Thus, an extraction protocol was first optimized to determine the total phenolic compounds and the antioxidant activity of CM. The aqueous CM antioxidant extract was then incorporated into cellulose acetate (CA) film-forming solution in different concentrations (1, 3 and 5 wt.%) to be further coated in corona-treated LDPE to obtain LDPE/CA-CM bilayer systems. CA and CA-CM film-forming solutions were successfully coated onto the surface of LDPE, showing good adhesion in the final bilayer structure. The optical, microstructural, thermal, mechanical and oxygen barrier performance, as well as the antioxidant activity, were evaluated. The active coating casted onto the LDPE film did not affect the high transparency of LDPE and improved the oxygen barrier performance. The antioxidant effectiveness of bilayer packaging was confirmed by release studies of *Cucumis metuliferus* from the cellulose acetate layer to a fatty food simulant. Finally, the LDPE/CA-CM active materials were also tested for their application in minimally processed fruits, and they demonstrated their ability to reduce the oxidation process of fresh cut apples. Thus, the obtained results suggest that CA-CM-based coating can be used to easily introduce active functionality to typically used LDPE at industrial level and enhance its oxygen barrier, without affecting the high transparency, revealing their potential application in the active food packaging sector to extend the shelf-life of packaged food by prevention of lipid oxidation of fatty food or by prevention fruit browning.

## 1. Introduction

Increasing ecological concern aimed towards a reduction of the environmental impact of short-term plastics (i.e., packaging, disposable cutlery, agricultural mulch films, etc.) is contributing to a move towards a circular economy model, in which a more sustainable plastic industry is continuously developing. The deliberate introduction of bio-based and biodegradable plastics in the field of packaging will make it possible to reduce the consumption of non-renewable petrochemical-based resources, as well as to prevent the accumulation of plastics waste in the environment (i.e., landfills, oceans, etc.) within the frame of the circular economy [1,2].

Cellulose acetate (CA) is a thermoplastic biodegradable polymer extensively studied for packaging applications owing to its excellent optical transparency and high toughness, and because it has the advantage of being produced primarily through the esterification of the renewable and most abundant polymer in nature: cellulose [3,4,5]. In fact, among cellulosic derivates, CA is extremely attractive for the packaging field mainly because of its low price, good biodegradability and non-toxicity, as well as due to its having a better processability than cellulose, as it can be processed either by solvent-casting, melt-blending or electrospinning approaches [6,7,8]. Moreover, CA has been widely used for the development of active packaging materials by means of the incorporation of active compounds (i.e., antioxidant and antimicrobial substances) into the CA polymeric matrix [9,10,11]. Active food packaging has advantages over direct addition of active compounds into the foodstuff, such as the use of additives in lower concentrations, as well as extension of shelf life due to the controlled release of active compounds during storage [12,13]. Moreover, it has an important effect on the reduction of deterioration reactions that begin at the food surface due to a more significant interaction with the surface of the packed food [11,14,15]. Additionally, there is a growing trend in the food packaging industry to replace synthetic additives with natural antioxidants in both petrochemical-based [16,17,18,19] and bio-based and biodegradable polymers [10,20,21,22], particularly with tocopherol, catechols, essential oils, and plant extracts [16,20,23].

In this regard, coatings are progressively becoming more widely recognized as a powerful tool for extending the shelf life of food by improving many properties of plastic materials as well as a simple method for providing specific active functions (e.g., antioxidant, antimicrobial, etc.) to the final food packaging [24]. In fact, the addition of active compounds into food coatings or packaging coatings has some advantages, as they act only at the surface level, and can be applied at any stage of the food supply chain [13,21]. In the food packaging sector, coating technology represents the most efficient and affordable solution for attaining high barrier properties against oxygen for light packaging, particularly in the case of polyolefins (i.e., polypropylene (PP) and polyethylene (PE)) [25]. In this regard, the application of CA-based active coatings on a typical film packaging material (e.g., low-density polyethylene (LDPE)) is an advantageous alternative for easily providing the final material with specific active performance. In fact, LDPE is one of the most widely consumed polymers in the food packaging field; it is extensively used in film to cover foodstuff due to its low cost, high resistance to tearing, low heat seal temperature, and high water barrier, as well as its high production efficiency [2,19,26]. However, it presents high oxygen permeability, which is a crucial property for plastic food packaging films [27]. Several strategies have been explored to improve the LDPE barrier performance for food packaging applications through blending [28,29], development of nanocomposites [30,31], or by using multi- or bi-layer approaches (i.e., surface coatings, sandwich structures, electrospun deposition, etc.) [25,32,33,34,35]. Coating approaches are of high interest since they make it possible to obtain a bi-layer structure using an easy, scalable and cost-effective method at an industrial level. Thus, applying a CA-CM layer to commercially available LDPE films by a simple coating process has the potential to reduce the oxidation process of packed food, providing the CA with better intrinsic oxygen barrier performance than LDPE, as well as offering the additional advantage of giving active packaging technology to the final formulation by simply incorporating antioxidant compounds into the CA-based film-forming solution.

*Cucumis metuliferus* (Cucurbitaceae) is an annual climber plant, native to Africa, that grows specifically from South Africa to tropical Africa [36]. It is known for its potential benefits to human health, and it has been suggested that it possesses antifungal, antimicrobial, antiviral and antioxidant effects, as well as chelating power [37,38]. It is called African homed melon, jelly melon and kiwano. The commercial culture of *C. metuliferus* began in New Zealand, where it was commercialized as an exotic fruit during the eighties. The main commercial advantages of *C. metuliferus* are that it grows rapidly and remains in good condition for around 6 months without cold storage [39,40]. For this reason, the commercialization was extended, and nowadays it grows in New Zealand, Australia, Chile, Argentina, Venezuela, Spain, Portugal, Germany, Italy, Israel and California [37,40]. Young *C. metuliferus* fruit is dark green with mottled light green spots, while as it ripens it becomes bright orange with very sharp spines [36]. In the interior is a mass of green, translucent, slightly mucilaginous juice-sacs enclosing many tightly packed, flat seeds [39]. Although the antioxidant ability of *C. metuliferus* has been determined [36,38], to the best of our knowledge its use in the development of antioxidant active packaging coating has not yet been proposed.

The main goal of the present research work was to assess the potential production of antioxidant active coatings for food packaging proposes based on cellulose acetate loaded with *C. metuliferus* fruit extract (CM). Initially, the extraction of antioxidant agents from *C. metuliferus* fruit was optimized by evaluating extraction procedures using different solvents: water, ethanol and ethanol 50%. Then, the obtained extract was incorporated into a cellulose acetate solution in different proportions (1, 3 and 5 wt.%). While coatings require substrates with high surface energy, LDPE is known to possess low surface energy as a consequence of its non-polar nature. Hence, LDPE is frequently surface treated to promote good adhesion between the polyolefin and the coating [33,41]. Thus, the obtained CM-functionalized CA film-forming solutions were coated onto commercial LDPE films. The obtained bi-layer structures were fully characterized considering the intended use in the active food packaging field. Thus, the correct adhesion of CA coating into corona-treated LDPE film was corroborated by scanning electron microscopy (SEM). The effect of the CA-based coating on the optical properties of LDPE was investigated by UV-visible measurements and the determination of colorimetric properties in the CIELab space. The mechanical and barrier performances were also evaluated with the aim of assessing their suitability for the food packaging sector. Finally, since these materials are intended for active food packaging applications, the release ability of the antioxidant compounds of *C. metuliferus* fruit from bilayer materials was analyzed in a fatty food simulant, as well as in direct contact with fresh-cut apples in order to get information of the possible application of these sustainable materials at an industrial level intended for both fatty food and fresh fruit protection.

## 2. Materials and Methods

### 2.1. Materials

Cellulose acetate with Mn = 30,000 and 39.8 wt.% acetyl content (CA degree of substitution = 2.5 [42]) was supplied by Sigma-Aldrich (Santiago, Chile). A commercial corona-treated low-density polyethylene (LDPE) film was kindly supplied by EDELPA (Santiago, Chile). The *Cucumis metuliferus* fruits were obtained at a local market in Santiago de Chile. 2,2-azinobis(3-ethylbenzothiazoline-6-sulphonate) (ABTS), Folin Ciocalteu phenol reagent, anhydrous sodium carbonate, gallic acid and 6-hydroxy-2,5,7,8-tetramethylchroman-2-carboxylic acid (Trolox) were purchased from Sigma Aldrich. Acetone (99.9% HPLC grade) and absolute ethanol (99.9% HPLC grade) were supplied by Merck (Santiago, Chile).

### 2.2. Methods

#### 2.2.1. *Cucumis metuliferus* Extraction Optimization

**Figure 1:**
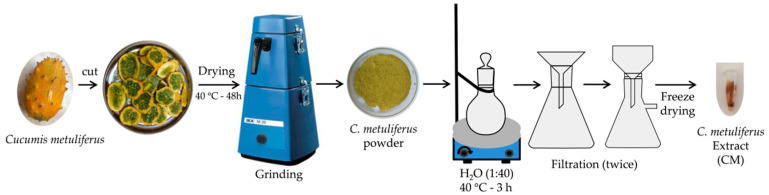
Schematic representation of antioxidants extraction procedure from *Cucumis metuliferus* fruit.

The *C. metuliferus* fruits were cut into slices and dried at 40 °C for 48 h. They were mechanically grinded to obtain powder by means of a cutter grinder. With the aim of obtaining the CM extract with highest antioxidant performance, it was extracted from *C. metuliferus* fruit powder using three different solvent systems: water, ethanol and 50% aqueous ethanol (v/v, EtOH 50%). Approximately 500 mg of *C. metuliferus* fruit powder was dispersed in 30 mL of each solvent and vigorously stirred for 180 min at 40 °C. The obtained viscous extracts were then filtered twice and used for the determination of radical scavenging activity and the measurement of total phenolic content (PC). Figure 1 show the schematic representation of *C. metuliferus* extract (CM) extraction procedure from *Cucumis metuliferus* fruit.

The total phenolic content (PC) of the *C. metuliferus* extract was colorimetrically determined by means of the Folin-Ciocalteu method according to the methodology adapted in previous work by Lopez de Dicastillo et al. [10]. In brief, 0.2 mL of Folin-Ciocalteu reagent and 3.1 mL of distilled water were mixed with 0.1 mL of each CM extract and kept in darkness for 5 min. Then, 0.6 mL of anhydrous Na_2_CO_3_ 20% (w/v) was added, shaken and then kept in the dark for 2 h. The PC was determined by means of the absorbance at 765 nm in a UV-vis spectrophotometer. The measurements were performed in triplicate and the results were expressed as mg gallic acid equivalent (GAE) per 100 g of dried sample.

The evaluation of the antioxidant ability of *C. metuliferus* fruit extract in the three different solvents assayed was determined by means of Ferric Reducing Antioxidant Power (FRAP), as well as radical 2,2′-azinobis(3-ethylbenzothiazoline-6-sulphonate (ABTS^·+^) methods, since they are simple, inexpensive and robust techniques [10,43]. FRAP follows a Single Electron Transfer (SET) method, and thus detects the ability of the antioxidant to transfer one electron to deactivate the reactive functional group of ferric 2,4,6-tripyridyl-s-triazine (TPTZ) [12]. In fact, FRAP measures the reduction to a blue ferrous product, which is absorbed at 593 nm [12,44]. Meanwhile, the ABTS method monitors the inhibition of oxidation of a suitable substrate, which may be neutralized either by direct reduction via SET or by radical quenching via Hydrogen Atom Transfer (HAT) [12,44]. HAT-based methods measure the antioxidant ability to quench free radicals by hydrogen donation [44]. The inhibition of the cationic radical ABTS^·+^ due to the presence of antioxidant compounds from *C. metuliferus* fruit extract was followed by the reduction of the characteristic wavelength absorption spectrum at 715 nm [16,45]. FRAP and ABTS methods were performed in triplicate, and results were expressed as Trolox equivalents per 100 g of fruit sample.

#### 2.2.2. Preparation of *Cucumis metuliferus*-Loaded Cellulose Acetate-Coated LDPE Films

**Figure 2:**
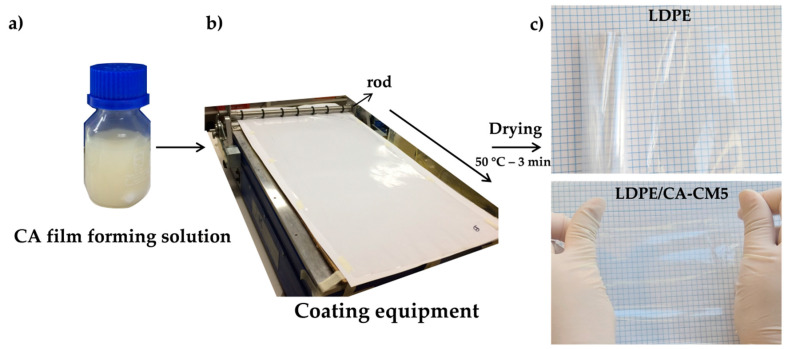
Schematic representation of the bi-layer film-forming process: (**a**) film coating solution, (**b**) coating application over LDPE film followed by drying process, and (**c**) visual appearance of the CA-CM coated LDPE films.

Cellulose acetate (CA) coating was prepared by dissolving 6 g of CA in 40 mL of acetone (0.15 g/mL) at 50 °C under stirring. The antioxidant cellulose acetate coating (CA-CM) was prepared by adding 1, 3 and 5 wt.% of the *C. metuliferus* antioxidant extract (CM) respect to CA weight (Figure 2a).

Each acetate film-forming solution was poured onto the corona-treated side of LDPE films with a lab-scale automatic applicator (Multicoater RK Printcoat, model K303, Royston, UK) equipped with a steel horizontal rod to obtain a homogeneous wet coating material of 80 µm (speed of 5 m/min) and at room temperature (Figure 2b). The coated LDPE films were immediately dried at 50 °C for 3 min and transparent films were obtained (Figure 2c). CA-CM films were also prepared for comparison. Thus, the film-forming solutions were casted on a 50 mm-diameter Petri dish and dried at 50 °C for 3 h.

Figure 2 schematically summarizes the bi-layer structure film preparation procedure, starting from the film-forming solution (Figure 2a), its application onto the LDPE film surface with the automatic applicator (Figure 2b), and the resulting bilayer LDPE film coated with CA-CM (Figure 2c).

#### 2.2.3. Film Characterization

##### Characterization of *Cucumis metuliferus*-Loaded Cellulose Acetate-Coated LDPE Films

The thickness of the obtained bilayer films was measured with a Digimatic Micrometer Series 293 MDC-Lite (Mitutoyo, Tokyo, Japan) at ten random positions over the film surface.

The absorption spectra in the 700–250 nm region of bilayer films were obtained using a Perkin Elmer (Lambda 35, Waltham, MA, USA) UV-VIS spectrophotometer.

The color properties of the films were measured in the CIELab space in a Minolta colorimeter CR-410 Chroma Meter (Minolta Series, Tokyo, Japan). The colorimeter was calibrated with a white standard tile. Measurements were carried out in quintuplicate at random positions over the CA-based coating surface layer of the LDPE films and average values for these five tests were used to calculate the total color differences (ΔE) induced by the presence of CA and CA-CM coatings into LDPE by means of Equation (1):

(1) $$\Delta E=\sqrt{{\left(\Delta a^{*}\right)}^{2}+{\left(\Delta b^{*}\right)}^{2}+{\left(\Delta L\right)}^{2}}$$

where a*, b* and *L* are the color coordinates *L* (lightness), a* (red-green) and b* (yellow-blue).

The cross cryo-fractured surface microstructure of the cross-section of the bi-layer structures was observed by scanning electron microscopy (SEM) using a JEOL F-6335 microscope. Samples were previously sputtered with a gold layer to make them conductive.

Thermogravimetric measurements were carried out in a Mettler Toledo Gas Controller GC20 Stare System TGA/DCS thermal analyzer (Schwerzenbach, Switzerland). The experiments were conducted under dynamic mode and under nitrogen atmosphere (flow rate of 50 mL/min). Film samples were heated from room temperature to 700 °C at 10 °C/min. The initial degradation temperatures (T_0_) were determined at 5% mass loss. Meanwhile, the temperatures at the maximum degradation rate (T_max_) were calculated from the first derivative of the TGA curves (DTG) for CA (T_maxCA_), as well as for LDPE (T_maxLDPE_).

The mechanical properties of the LDPE and LDPE/CA bilayer films were determined by tensile test measurements at room temperature with an IBERTEST ELIB 30 (S.A.E. Ibertest, Madrid, Spain) machine equipped with a 100 N load cell. Tests were performed in rectangular strips (dimensions: 100 × 10 mm^2^), initial grip separation of 50 mm and crosshead speed of 2 mm/min. Five different samples were tested, and average values of tensile strength and elongation at break were reported.

The oxygen permeation rates of the LDPE and LDPE/CA films were determined at 23 °C and 0% relative humidity (RH) by means of an OXTRAN MOCON model 2/21 ML (Lippke, Neuwied, Germany). Films were previously purged with nitrogen for a minimum of 16 h prior to exposure to an oxygen flow of 10 mL/min. The oxygen permeability coefficient (OP) is proportional to oxygen transmission rate per thickness, OTR*e (e = thickness, mm), and thus, the OTR*e values were used to compare the oxygen barrier properties of the films.

Release studies of the active compounds from the CA-CM coated LDPE films were conducted by immersion of the films into a fatty food simulant (simulant D1 = solution of 50% ethanol) at 40 °C for 10 days [46]. Double sided, total immersion migration tests were carried out by total immersion of 3 cm^2^ piece of each film in 5 mL of food simulant (area-to-volume ratio = 6 dm^2^/L) contained in a glass vial. Since ABTS^·+^ is an indicator radical that can be neutralized either by direct reduction via SET or by radical quenching via HAT [12], the antioxidant performance of the developed film formulations was measured by means of ABTS method. Therefore, the antioxidant activity of the *C. metuliferus* fruit extract released in the fatty food simulant was regularly analyzed by the scavenging activity of stable free ABTS^·+^ radicals, expressed as Trolox equivalents per film area.

The obtained CA-CM coated LDPE films were also tested as fresh fruit browning prevention systems. Thus, LDPE/CA and LDPE/CA-CM films were used to pack fresh-cut apples. Apples were previously washed with tap water, peeled and sliced with a clean knife and packed with the developed bilayer materials in direct contact with the CA-CM layer. The browning of apples was indirectly measured by colorimetrical measurements in the CIELab space at 30 °C for 92 h. The packed sliced apples color changes were measured in a Minolta colorimeter CR-410 Chroma Meter (Minolta Series, Tokyo, Japan). The colorimeter was calibrated with a white standard tile. Measurements were carried out in quintuplicate at random positions over the packed apple surfaces and average values for these five tests were used to calculate the total color differences (ΔE) by Equation (1).

Significant differences in the determination of PC as well as in the assessment of antioxidant activity of *C. metuliferus* fruit extract (FRAP and ABTS methods) were statistically calculated by one-way analysis of variance (ANOVA) with OriginPro 8 software using Tukey’s test with a 95% confidence level. Similarly, for bilayer films the colorimetric coordinates determinations, tensile test measurements, the release studies of CM-CA-coated LDPE films, as well as the color changes measurements in packed sliced apples, were also statistically calculated by one-way analysis of variance (ANOVA) with OriginPro 8 software using Tukey’s test with a 95% confidence level.

## 3. Results and Discussion

### 3.1. Antioxidant Activity of Fruit Extracts

The antioxidant ability of natural extracts is highly dependent on the chemical structure of the active compounds, as well as on the mechanisms used (SET and/or HAT) [12]. Therefore, there is no general standardized method for the extraction of antioxidant agents from heterogeneous systems, such as foods and crops [10]. With the aim of evaluating the most effective extraction process, various solvent systems were assayed based on previous works [43,47]. Table 1 reports the polyphenolic content (PC), as well as the antioxidant activities measured by two methods: FRAP (which operates by the SET mechanism) and ABTS (which operates by both the HAT and SET mechanisms) of the resulting fruit extracts. The lowest polyphenolic extraction efficiency was obtained for pure ethanol, while PC and antioxidant power values of aqueous and aqueous/ethanol extractive solutions did not present significant differences. Matsusaka et al. studied the PC of edible (pulp) and non-edible (seed and peel) parts of *C. metuliferus* from Japan, extracted in EtOH 50%, and similar values were obtained [38]. The peel and seeds showed higher phenolic content than the edible pulp [38].

With respect to antioxidant ability, it is known that the FRAP method is more specific for hydrophilic antioxidants, while ABTS is a good method for evaluating both lipophilic as well as hydrophilic antioxidants [48]. The FRAP and ABTS methods (Table 1) indicated that FRAP values were higher for aqueous and 50% ethanolic extractive solutions (without significant differences, *p* > 0.05) than for ethanol (*p* < 0.05). Matsusaka et al. also determined the radical scavenging activity using the ABTS method, obtaining around 200 µmol Trolox/g of whole fruit (edible and non-edible parts), which is approximately 5 mg GAE/100g fruit, which is lower than the results obtained here. Motlhanka studied the antioxidant performance of aqueous, methanolic and under chloroform *C. metuliferus* extracts (pulp and skin) using the DPPH method, and the results indicated that aqueous extract exhibited the strongest antioxidant response, while methanolic extract possessed moderate antioxidant response and low activity in chloroform [36]. Both works were in accordance in confirming that principal phenolic compounds were mainly extracted by using distilled water. Although the chemical composition of *C. metuliferus* has aroused little scientific interest, it is known that the pulp contains beta carotene and vitamins A (retinol), B (B_1_–B_3_, B_5_, B_6_ and B_9_) and C [37]. Meanwhile, the seeds are rich in linoleic acid [49], α-tocopherol and γ-tocopherol [37,49], lipases, lipoxygenases enzymes [49] and inorganic ions, such as potassium, calcium, iron, magnesium, phosphorus and zinc [37,49]. It has been reported that the fruit also comprises alkaloids, carbohydrates, cardiac glycosides, flavonoids (i.e., rutin, miricetin and quercetin), saponins, tannins, steroids and terpenoids [37,40]. Although PC values manifested clear differences between extracts, the results concluded that extracts were rich in both hydrophilic antioxidants (as determined by FRAP and ABTS), and lipophilic antioxidants (as determined by ABTS). In fact, the antioxidant activity determined by FRAP showed the lowest values for pure ethanol (*p* > 0.05) and higher values for water and aqueous/ethanol extractive solutions, without significant differences between the water and aqueous/ethanol extractive solutions (*p* > 0.05), in accordance with the PC results. This fact was probably because flavonoids, which are generally more soluble in ethanol, can be bonded with saccharide groups, which are more water soluble, as has already been reported in previous work [43]. The correlation between PC and FRAP values between extracts occurred principally because the PC method is based on the oxidation of phenols by a molybdotungstophosphoric reagent through single electron transfer [43]. On the other hand, the ABTS values of extracts did not present significant differences. Non-glycoside phenolic compounds such as flavanol and flavones generally present better solubility in alcoholic extractive solutions. These PCs were probably molecules with higher chemical composition where a simple molecule is able to scavenge several radical molecules and whose antioxidant activities through the HAT mechanism were also taken into account [43,44,50,51,52]. Due to the obtained results (Table 1), and considering environmental aspects in evaluating the extractive effectiveness, water was selected for the extraction procedure, which is schematically represented in Figure 1. In brief, dried *C. metuliferus* fruit was mechanically grinded to obtain a powder and dispersed in distilled water, which was then heated (40 °C, 180 min at 500 rpm). The resulting solution was filtered twice (double ring qualitative filter paper GE, Grade fast 101), frozen and then the extract was concentrated to dryness by means of a freeze drying process. The obtained viscous extract was then used for the preparation of antioxidant coatings. 

### 3.2. Coating Process for Bilayer Film Forming

*C. metuliferus* extract (CM) was incorporated at three different concentrations (1, 3 and 5 wt.%) into cellulose acetate solution for the development of active coating film-forming solutions. It is widely known that a polymer should be soluble in a solvent with a similar solubility parameter (δ), and thus δ represents an important parameter when working with polymeric solutions [8,14,42]. Thus, good solubility of CA into acetone is ascribed to their close solubility parameters, which are between 19.6 and 25.1 MPa^1/2^ [8,42,53] for CA and between 19.9 and 20.1 MPa^1/2^ [8,53] for acetone. Concerning the solubility parameters of the main components of CM which are 18.9 MPa^1/2^ for betacarotene [54], 18.7 MPa^1/2^ for retinol [55], 20.2 MPa^1/2^ for α-tocopherol and 20.3 MPa^1/2^ for γ-tocopherol [54], good miscibility between CA and CM should be expected. 

It is known that the viscosity of a polymeric solution greatly depends on the polymer concentration [56], thus it can be regulated by simple varying the polymer concentration in the film-forming solution. Therefore, 6 g of CA powder was firstly dispersed in 40 mL of acetone under continuous stirring at 50 °C, until complete dissolution [42]. Since in this work CM was obtained by means of an aqueous extraction procedure, it should be taken into account that low amounts of residual water can act as a non-solvent, which can potentially compete against the interactions between CA and acetone [42]. In fact, the solubility parameter of water is 47.9 MPa^1/2^ [8,42], and thus the solubility parameter of solvent will increase as the presence of water increases in the acetone:water system. From semi-dilute to concentrated polymeric solution, the polymer dimensions decrease until critical concentration (c^+^) is reached, at which point they shrink to their unperturbed dimensions and remain constant [57]. Necula et al. studied several polymeric solutions of CA in acetone 95% *v/v* at different concentrations up to 0.4 g/mL, at different temperatures (from 20 to 50 °C). CA critical concentration at which the polymer coils begin to overlap with each other (0.013 > c* > 0.018 g/mL), as well as the critical concentration for reaching the unperturbed state, c^+^ (0.098 > c^+^ > 0.142 g/mL) (c^+^ ≅ 8 c*), were determined [58]. Thus, in the present work, in order to ensure that the cellulose acetate coils in acetone (or acetone with low amounts of water as non-solvent, i.e., acetone > 90%) were able to contract toward the unperturbed size state, the selected concentration of CA and/or CA-CM film-forming solution was 0.15 g/mL.

Due to the non-polar nature of LDPE for coating applications and for effectively formation of adhesion joints, it needs a previous surface treatment [41]. Thus, a commercial corona-treated LDPE was selected as substrate in order to increase the poor adhesion properties of LDPE. It should also be taken into account that mass and/or heat transfer takes place, and the polymeric systems become thermodynamically unstable during the solvent coating process, and therefore, phase separation can take place [42]. The molecules of CA in acetone (boiling point 56 °C) are characterized by high chain rigidity, but the chain stiffness decreases as temperature increases and, as a result, their flexibility increase [59]. Thus, in order to select the coating drying conditions, the temperature was increasingly varied within the temperature range from 45 °C to 50 °C and the time was varied between 2 and 3 min through trial-and-error practice until bilayer films with good-quality visual appearance were obtained. That is, the coating parameters were adjusted until a homogeneous solution coating completely covered the LDPE film without apparent phase separation. The processing drying temperature and time of CA and/or CA-CM coated onto the LDPE films were 50 °C and 3 min, since these processing parameters made it possible to obtain transparent films without visual defects (Figure 2c). The obtained film thicknesses ranged from 50 ± 2 μm to 63 ± 3 μm, confirming the low thickness of the CA coating in the final bilayer formulation. All LDPE/CA-coated film formulations were transparent without affecting the high transparency of the LDPE (see upper image in Figure 2c), even at the highest *C. metuliferus* fruit extract concentration of 5 wt.% (see lower image of LDPE/CA-CM5 in Figure 2c).

### 3.3. Optical and Morphological Properties

**Figure 3:**
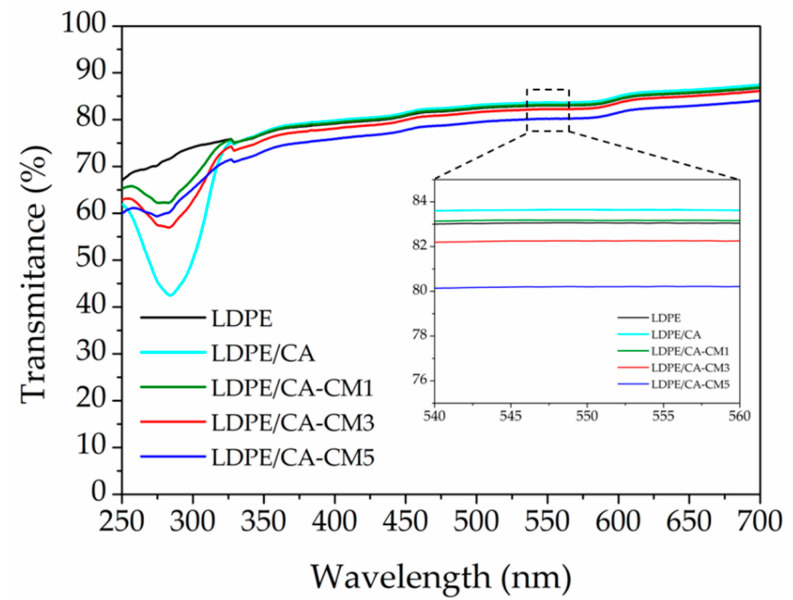
UV-vis spectra of LDPE and LDPE/CA-coated films.

The processing conditions used here made it possible to obtain transparent and thin bilayer films (see thickness in Table 2). It should be highlighted that transparency is one of the most important characteristics of the polymeric films for food packaging. Thus, these results were confirmed by means of the determination of their optical properties (Figure 3). No significant differences were observed on the light transmission along the visible region of the spectra (400–700 nm), suggesting that CM was homogeneously dispersed over the CA matrix. The transparency of the LDPE/CA films was measured in the range 540–560 nm (see zoom in Figure 3). The addition of CA had practically no effect on the high transparency of the LDPE. Similarly, the incorporation of CM into the CA matrix had practically no effect on the high transparency of the LDPE/CA, particularly when it was added at low amounts such as 1 wt.% and 3 wt.% (LDPE/CA-CM1 and LDPE/CA-CM3). Meanwhile, the incorporation of the highest amount of CM (5 wt.%) produced a slight reduction in the transparency of the LDPE/CA (see zoom Figure 3), but high transparency was still observed, as can be seen in the visual appearance of this bilayer film (see as example the lower image of LDPE/CA-CM5 in Figure 2c). With respect to the UV spectra region (250–400 nm), the LDPE/CA film showed a reduction of the transmittance of LDPE due to the fact that CA absorb light in the region below 250 nm. This absorption was slightly reduced with increasing amounts of CM due to the decreasing CA content in the formulation.

The color parameters of films were measured in the CIELab space (Table 2). All materials showed high lightness values. The CA and CA-CM coating application did not produce significant changes in *L* values, which is in good accordance with the high transparency observed for the visual appearance of the films (Figure 2c). The negative values obtained for the a* coordinate are indicative of deviation towards green color, but these values were very close to zero. This coordinate decreased particularly in the LDPE/CA-CM5 film with the highest amount of CM, showing significant (*p* > 0.05) differences with respect to LDPE/CA-CM materials with lower amounts of CM (LDPE/CA-CM1 and LDPE/CA-CM3 films). Meanwhile, no significant differences in the b* coordinate were observed between LDPE/CA-based films with respect to the LDPE film, with the exception of LDPE/CA-CM5 film, which showed significant differences (*p* > 0.05) towards positive values, which are indicative of deviation towards yellow color. Similarly, the highest color differences with respect to uncoated LDPE film were observed for LDPE/CA-CM5. Nevertheless, it should be highlighted that all formulations showed lower ∆E values than 0.3, being considerably lower than ∆E of ± 2.0, which is the value typically considered to be the threshold of perceptible color difference for the human eye [13], and even lower than ∆E of ± 0.5, which is the total color difference able to be recognized by a sensorial panel [60].

**Figure 4:**
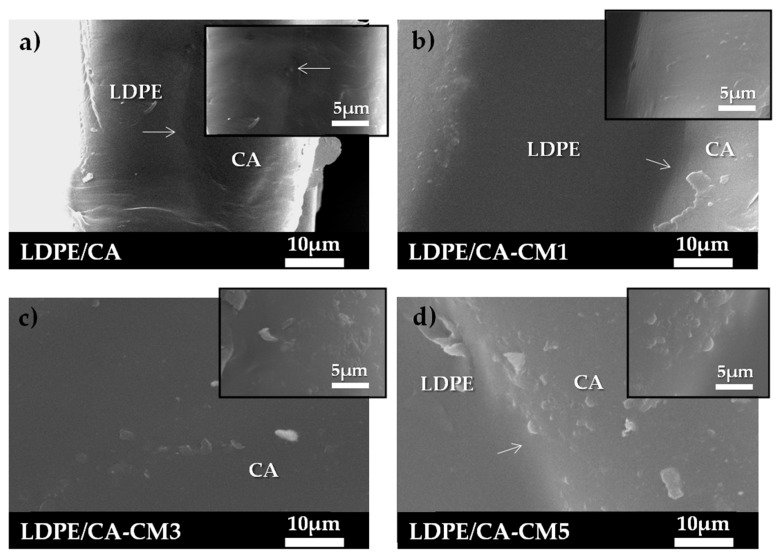
SEM of cross-fracture surface: (**a**) LDPE/CA, (**b**) LDPE/CA-CM1, (**c**) LDPE/CA-CM3, and (**d**) LDPE/CA-CM5. 2000× (inset figures 5000×).

The morphological structure of polymeric films is an essential characteristic, since it directly affects the mechanical and barrier performance of the final materials, particularly important in the packaging sector, where it can ultimately influence the commercial success. The adhesion between polymeric layers in multilayer systems is frequently evaluated by observing the microstructure of the materials using microscopic techniques [21,61]. Figure 4 shows the micrograph of the cross-section surfaces of LDPE-, LDPE/CA- and LDPE/CA-CM-based films analyzed by SEM. The SEM analysis was carried out to evaluate the morphological investigation of the bilayer structures, as well as to evaluate the effect of active films on the microstructure at the different concentrations of CM (1, 3, and 5 wt.%) with respect to the CA polymeric matrix used to produce the different LDPE/CA-CM-based formulations. In the SEM micrographs, both polymeric layers can be clearly distinguished (see arrows), showing very good adhesion, with no detachment being observed, revealing that cellulose acetate had been successfully coated onto the surface of corona-treated LDPE (Figure 4a). The LDPE layer presented the typical smooth surface of LDPE in all bilayer formulations [60]. In LDPE/CA film, the CA layer presented a homogenous structure without the presence of pores on the coating structure, suggesting that no pores were formed during the process as a consequence of the acetone evaporation, as can occur in CA-based film processed by solvent casting [62]. This result confirmed the success of the coating process developed here in which the CA/acetone ratio used, as well as the drying conditions (50 °C during 3 min), are crucial. The addition of CM into CA coating film-forming solutions did not affect the adhesion of either polymeric layer (Figure 4b–d). However, some compact rougher structures were observed with increasing amounts of CM in the CA layer, which was particularly evident in the LDPE/CA-CM5 film (Figure 4d). This behavior can be ascribed to interaction among active components that tend to agglomerate at high concentrations.

Although further studies should be performed to ensure the good adhesion between CA-CM coating and corona-treated LDPE substrate (e.g., sealability, as well as friction and scratch resistance), optical and morphological SEM analysis of bilayer systems revealed that good adhesion had been achieved through the applied drying process parameters (time and temperature) immediately after coating CA-CM film forming solution onto LDPE film. In fact, on one hand, CA-CM coating layer had practically no effect on the LDPE substrate transparency, while on the other hand the absence of porous structures and/or phase separation in SEM images suggests good adhesion between both layers.

### 3.4. Thermal Properties

**Figure 5:**
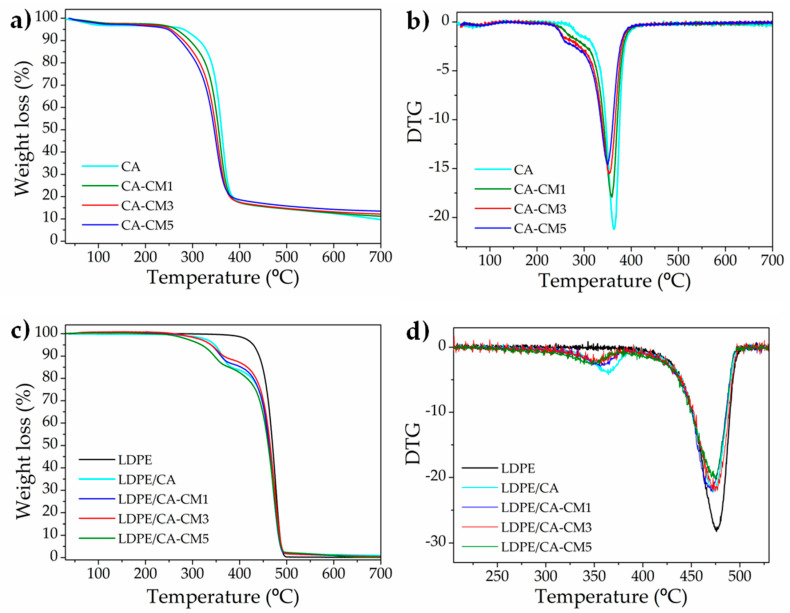
Thermogravimetric analysis: (**a**) TGA of CA-based layer, (**b**) DTG of CA-based layer, (**c**) TGA of LDPE/CA-based bilayer, and (**d**) DTG of LDPE/CA-based bilayer.

With the aim of studying the thermal degradation of each layer, CA and CA-CM-based formulations were prepared by solvent casting, and the TGA parameters, as well as the residue at 700 °C, are summarized in Table 3. Meanwhile, the TGA and DTG curves are shown in Figure 5a,b, respectively.

A small mass loss below 130 °C belonging to the volatilization of the volatile matter and/or to the evaporation of absorbed and bound water was seen in all CA-based films [6,60]. Subsequently, there was a thermal degradation (from around 180 to 300 °C) related to the loss of acetyl groups, followed by acetic acid volatilization, which could further catalyze the decomposition of cellulose [63]. Next, the two typical thermal degradation processes of cellulose acetate were also observed, corresponding to the fragmentation of macromolecular structure of the cellulosic chain (T_maxCA_ = 364 °C), followed by the last thermal degradation step, which starts at around 450 °C, belonging to the carbonization of products (≈550 °C) to ash [6,60]. Neat CA film still yielded small residual ashes after degradation at 700 °C (less than 10%), since CA requires higher temperatures in order to achieve practically no residue (790 °C) [63]. The incorporation of *C. metuliferus* extract reduced the thermal stability of cellulose acetate matrix by reducing the onset degradation and maximum degradation temperatures to lower values. After 700 °C, the residual ashes for the CA-CM samples were somewhat higher, probably consisting of: (i) positive interaction between CM components and cellulosic structures formed during degradation (i.e., hydrogen-bonding interaction) that delayed the end of the main degradation step of the cellulose structure, (ii) lignocellulosic structures extracted from CM (lignin degradation takes place in a wide range of temperature, from 100 to 900 °C [64]), and/or (iii) inorganic components of CM.

The effect of the addition of the CA coating onto LDPE film was also investigated by TGA (Figure 5c,d). The addition of the cellulose acetate coating layer reduced the high thermal stability of LDPE, since CA presented lower thermal stability than LDPE. Thus, the onset degradation temperature was shifted approximately 80 °C toward lower values, from 429 °C in LDPE to 347 °C in the LDPE/CA film, while the T_max_ of LDPE was slightly reduced or largely maintained. 

The effect of the addition of CA-CM coating produced a similar behavior, and both the T_0_ and T_max_ of CA shifted to lower values, following the same tendency as that of the CA films (Table 3). Meanwhile, the T_max_ of LDPE was not affected by the presence of the CA-CM-based coatings. After 700 °C, the bilayer films presented practically no residue (less than 1%). Nevertheless, it should be highlighted that no significant degradation took place in the temperature region from room temperature to 200 °C, which is a considerably higher temperature than that at which the films are intended to be used during the food packing process, as well as during storage.

### 3.5. Mechanical and Oxygen Barrier Properties 

Films for food packaging are required to maintain their integrity with the aim of withstand the stress that occurs during shipping, handling and storage [1,2]. Thus, the mechanical properties of LDPE-, LDPE/CA- and LDPE/CA-CM-based films were studied by mean of tensile test measurements. Based on the tensile test results (Table 4), it seems that the mechanical properties of the coated LDPE films (LDPE/CA- and LDPE/CA-CM-based films) were controlled by the polyethylene layer. Nevertheless, it should be mentioned that multilayer films of plastic combined with biopolymers generally possess poor mechanical properties due to the poor mechanical strength of the biopolymers [65]. For instance, Shin et al. studied corn zein-coated LDPE, and their mechanical strength could not be measured due to the high brittleness of the corn zein layer, since it broke before the LDPE in the bilayer system [65]. In the present work, the LDPE/CA-based bilayer films exhibited a somewhat higher tensile strength, probably due to the composite structure and the higher tensile strength of CA polymeric matrix [5] with respect to that of LDPE [32], although without significance differences (*p* < 0.05). However, CA possessed very low elongation at break [5] and thus, it is probable that the very thin CA-based coating broke before the LDPE in the bilayer structure during the tensile test measurements. However, this was undetectable from the stress-strain curve (not shown) due to the very thin character of the CA layer. In fact, as Table 4 shows, it seems that the high flexibility of LDPE was not affected by the presence of the CA-CM coating (*p* > 0.05) in bilayer formulations. Moreover, comparing the LDPE/CA-CM-based films with respect to the LDPE/CA formulation, it seems that CM did not affect the mechanical performance of the LDPE/CA film, confirming the well dispersion of the *C. metuliferus* extract in the CA polymeric matrix, as was noted in SEM analysis (see Figure 4). Similar findings on the mechanical performance of LDPE coated with methylcellulose containing murta leaf (*Ugni molinae* Turcz) extract were observed in a previous work by Hauser et al. (2016). In that case, although the elongation at break of neat methylcellulose did not exceed 15%, high elongation at break (higher than 160%) in the bilayer structures was observed [32]. They ascribed this behavior to homogenous methylcellulose coating formation with good adhesion to the corona-treated LDPE [32].

One of the major challenges for coatings intended for LDPE is to increase the low oxygen barrier performance of this polymer. CA is recognized to have a higher barrier performance (OTR values around 650 cm^3^/m^2^ day [6]) than LDPE (LDPE film = 4750 650 cm^3^/m^2^ day, thickness = 0.05 mm). Thus, the application of a CA coating onto LDPE film drastically reduces the oxygen permeability, reducing the OTR*e values by between 19% and 31% (Table 4). The LDPE/CA-CM5 film presented slightly higher oxygen transmission values, probably due to its having the lowest homogeneity as a result of its high CM extract concentration. Although the oxygen barrier performance obtained here did not provide the final packaging material with a strong oxygen barrier performance, such as those provided by other polymeric matrices with well-known oxygen barrier performance (i.e., poly(ethylene terphthalate) (PET) with OTR*e < 3 cm^3^ mm/m^2^ day [4,66], EVOH which exhibits low OTR values under dry conditions with OTR*e < 4 cm^3^ mm/m^2^ day [67], or calcium and sodium caseinates with OTR*e < 7 cm^3^ mm/m^2^ day [13]), it showed the effectiveness of CA and CA-CM coating to improve these properties due to the good adhesion onto the corona-treated LDPE substrate. The improvement of the LDPE films’ barrier performance by coating it with different biopolymers such as whey protein [35], gelatin [66], chitosan or corn zein [65] has been already observed.

### 3.6. Antioxidant Activity of Active Bilayer Systems

**Figure 6:**
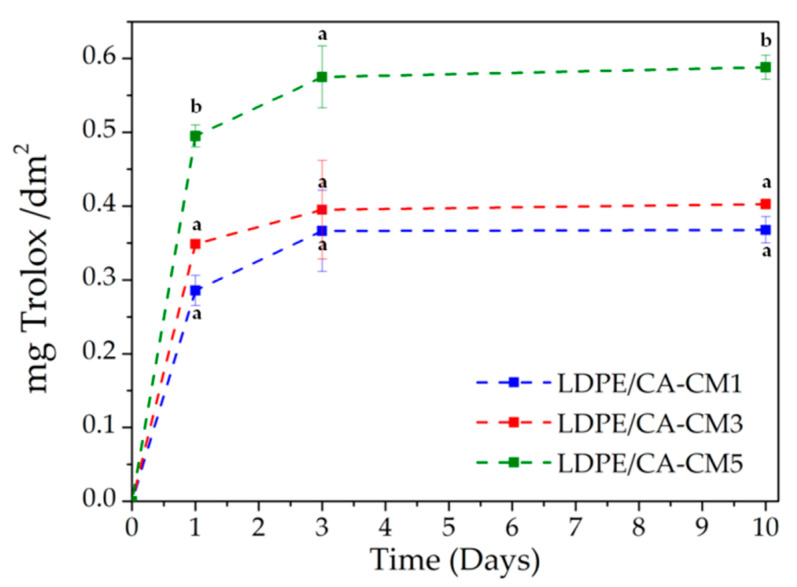
The *C. metuliferus* fruit extract antioxidant activity measured by ABTS method. ^a–b^ Different superscripts within the same day indicate significant differences between formulations (*p* < 0.05).

Considering that lipids are one of the main targets of oxidative reactions and, thus, lipid oxidation process responsible of a major problem in both natural and processed foodstuff [68], the antioxidant developed bilayer films were studied in direct contact with a fatty food simulant (simulant D1 = solution of 50% ethanol) [46]. Meanwhile, considering the complexity of different compounds in *C. metuliferus* fruit extract, the release studies of the active agents from the CA-CM coating layer of the LDPE films were indirectly measured through the determination of the total antioxidant activity into the food simulant, due to the fact that it is proportional to antioxidant release kinetics [10]. To evaluate both, lipophilic as well as hydrophilic antioxidants released, ABTS method was used and the measurements were performed after 1, 3 and 10 days in contact with. Non containing *C. metuliferus* fruit extract LDPE/CA film was also analyzed as control material and, as expected, did not show any ABTS radical scavenging activity (not shown). The antioxidant release kinetics indicated that more than 50% of active compounds were released during the first day (Figure 6). Subsequently, the release capacity moved on towards an equilibrium value on the third day in contact with the food simulant. The release kinetic of the active compounds of CM followed the second Ficks’ law of diffusion with an exponential growth to a maximum, in accordance with already reported works of active cellulose acetate films (i.e., CA loaded with ascorbic acid [69], L-tyrosine [69], thymol [70] and red onion extract [10]). As it was expected, the antioxidant activity increased with increasing amount of CM in the formulations. Thus, the higher antioxidant effectiveness was for the film with the higher amount of CM (LDPE/CA-CM5 film). However, higher antioxidant effect was observed in other cellulose acetate films such as CA loaded with 5 wt% or red onion extract which showed around 1 mg Trolox/dm^2^ film [10]. This result can be related with the lower PC and antioxidant performance of the *C. metuliferus* extract with respect to that of red onion. Nevertheless, it should be taken into account that the use of an active internal CA-based layer coated in an external LDPE layer may contribute to the effectiveness of antioxidant performance by slowing down the release rates and extending their action due to the interaction between both polymeric layers and the less exposition to the food simulant [61]. The major antioxidant ability of CM has been attributed to non-edible parts of the fruit (seed and peal) [38]. Thus, CM can result interesting not only for the development of antioxidant packaging materials, but also towards the use of this fruit waste from agri-food industry as a valorization resource of bioactive compounds giving an added value to the non-edible waste.

### 3.7. Anti-Browning Effect on Packaged Fresh-Cut Apple

**Figure 7:**
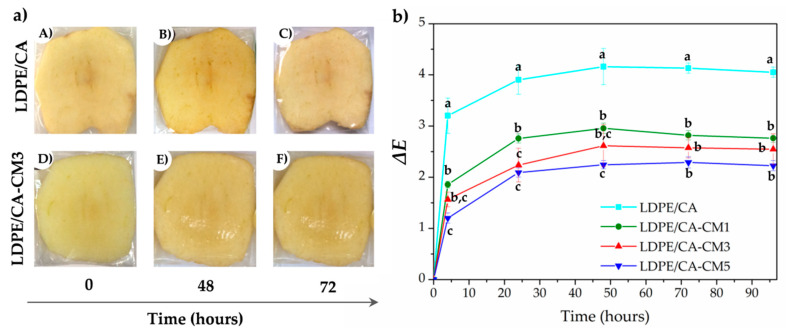
(**a**) Visual appearance of apples packed with LDPE/CA film: (**a**)/(**A**) immediately packed, (**a**)/(**B**) after 48 h and (**a**)/(**C**) after 72 h; and LDPE/CA-CM3 film: (**a**)/(**D**) immediately packed, (**a**)/(**E**) after 48 h, and (**a**)/(**F**) after 72 h packed; (**b**) Total color change evolution of fresh-cut apples packed with LDPE/CA and LDPE/CA-CM bilayer films over 96 h. ^a–c^ Different superscripts within the same day indicate significant differences between formulations (*p* < 0.05).

Another promising field of coating technology application is in the fresh and minimally processed fruit sector, which are highly perishable products [21]. Thus, the obtained active-coated LDPE films were also tested in the prevention of browning in fresh-cut apples (Figure 7). 

It is well known that fresh fruit browning is caused by enzymatic oxidation of phenolic compounds mediated by polyphenol oxidase activity, and two strategies for inhibiting this process are through the reduction of oxygen and addition of antioxidants [71]. Figure 7 shows the visual appearance of apple slices packed with non-active bilayer film (LDPE/CA, upper images in Figure 7b), as well as with active LDPE/CA-CM films, using LDPE/CA-CM3 as example (down images in Figure 7a) stored at 30 °C to simulate the worst foreseeable conditions. As can be seen, apples packed in LDPE/CA without *C. metuliferus* extract clearly exhibited a browning effect after 48 h (Figure 7a(B)) and 72 h (Figure 7a(C)). Meanwhile, this effect was less pronounced in LDPE/CA-CM-based formulations (Figure 7a(D–F)). These findings were corroborated by the determination of the evolution of color differences (∆E) of packed apples with all LDPE/CA-based bilayer formulations over 96 h of storage (Figure 7b). As expected, the highest color differences were observed in packed apples with un-functionalized LDPE/CA film Figure 7b, which was in good accordance with the visual browning observed in Figure 7a(B,C). Meanwhile, those packed fresh-cut apples containing CM in the coating layer showed less color difference, which decreased with increasing amounts of CM in the formulations (Figure 7b). In fact, LDPE/CA-CM5 formulation was able to reduce the ∆E value by around 50% with respect to the LDPE/CA film, reaching values around ∆E = 2.

## 4. Conclusions

Antioxidant compounds of *Cucumis metuliferus* (CM) fruit were successfully extracted by means of an aqueous extraction process and further incorporated into cellulose acetate (CA) matrix to develop antioxidant active coatings. CA was dissolved in acetone and CM was further added in concentrations of 1, 3 and 5 wt.%. The good miscibility of the film-forming solution was directly related to the fact that the main components of *C. metuliferus* show solubility parameters close to those of CA, as well as to acetone. The CA-CM-based film-forming solutions were successfully coated onto corona-treated LDPE films through a simple process. Very thin CA-CM layers were obtained, since the films’ thickness varied from 50 µm in the case of LDPE to thicknesses between 60 and 65 µm in the case of the bilayer LDPE/CA-CM films. SEM observations confirmed the proper adhesion of the CA coating onto the LDPE film for intended use as bilayer packaging materials. CA and CA-CM-based coatings induced a decrease in the thermal stability of LDPE, but exhibited enough thermal stability (T_0_ > 300 °C) for the intended use (i.e., during food packing or storage). The CA-CM-based layer provided improved oxygen barrier to LDPE film and did not affect its high transparency or colorlessness. Meanwhile, the CA layer containing different amounts of CM extract (1, 3 and 5 wt.%) showed its effectiveness as an antioxidant carrier, since CM either underwent a sustained release into a fatty food simulant, exerting free radical scavenging activity, or reduced the browning of fresh-cut apples in direct contact. Since the coating process proposed here is simple, and extremely flexible and low-cost, it is expected that the transfer of these active coatings from laboratory scale to industrial production will be easily feasible.

## Figures and Tables

**Table 1:** Total phenolic content (PC) and antioxidant measurements (ABTS and FRAP) of *C. metuliferus* extract in different solvents.

*C. metuliferus* Extract	PC (mg GAE/100g Fruit)	FRAP (mg Trolox/100g Fruit)	ABTS (mg Trolox/100g Fruit)
**H_2_O**	89.0 ± 5.1 ^a^	238.6 ± 8.6 ^a^	8.0 ± 0.2 ^a^
**EtOH**	47.2 ± 7.3 ^b^	161.3 ± 2.0 ^b^	9.6 ± 0.8 ^a^
**EtOH 50%**	101.5 ± 1.9 ^a^	241.1 ± 3.2 ^a^	9.0 ± 0.1 ^a^

^a–b^ Different superscripts within the same column indicate significant differences between formulations (*p* < 0.05).

**Table 2:** Color properties of CA-CM-coated LDPE films.

Formulations	Thickness (µm)	L	a*	b*	∆E
**LDPE**	50 ± 2	98.7 ± 0.1 ^a^	−0.03 ± 0.02 ^a,b^	2.02 ± 0.03 ^a^	- ^a^
**LDPE/CA**	55 ± 3	98.6 ± 0.1 ^a^	−0.02 ± 0.01 ^a,b^	2.05 ± 0.06 ^a^	0.05 ± 0.03 ^a,b^
**LDPE/CA-CM1**	60 ± 1	98.8 ± 0.3 ^a^	−0.01 ± 0.02 ^a^	2.04 ± 0.06 ^a^	0.10 ± 0.10 ^b^
**LDPE/CA-CM3**	60 ± 4	98.5 ± 0.2 ^a^	−0.04 ± 0.02 ^a^	2.09 ± 0.03 ^a^	0.18 ± 0.09 ^a,b,^
**LDPE/CA-CM5**	63 ± 3	98.5 ± 0.1 ^a^	−0.06 ± 0.01 ^b^	2.19 ± 0.02 ^b^	0.27 ± 0.03 ^b^

^a–b^ Different superscripts within the same column indicate significant differences between formulations (*p* < 0.05).

**Table 3:** TGA thermal properties of CA-CM-based films and LDPE/CA-CM-based films.

Formulations	T_0_ (°C)	T_maxCA_(°C)	T_maxLDPE_ (°C)	Residue at 700 °C (%)
**CA**	281	364	-	9.7
**CA-CM1**	265	360	-	11.2
**CA-CM3**	254	353	-	12.2
**CA-CM5**	247	351	-	13.5
**LDPE**	429	-	476	0.1
**LDPE/CA**	347	363	474	0.9
**LDPE/CA-CM1**	340	356	474	0.4
**LDPE/CA-CM3**	339	355	474	0.5
**LDPE/CA-CM5**	319	355	474	0.1

**Table 4:** Tensile test and oxygen barrier properties of LDPE/CA-CM-based films.

Formulations	Tensile Strength (MPa)	Elongation at Break (%)	OTR*e (cm^3^ mm/m^2^ day)
**LDPE**	8.5 ± 1.5 ^a^	455 ± 35 ^a^	237.7
**LDPE/CA**	9.7 ± 2.4 ^a^	480 ± 45 ^a^	170.7
**LDPE/CA-CM1**	9.5 ± 0.2 ^a^	455 ± 10 ^a^	164.2
**LDPE/CA-CM3**	8.1 ± 3.1 ^a^	420 ± 35 ^a^	171.6
**LDPE/CA-CM5**	8.7 ± 1.0 ^a^	475 ± 70 ^a^	193.2

^a^ Different superscripts within the same column indicate significant differences between formulations (*p* < 0.05).
